# *Clostridium difficile* in Retail Ground Meat, Canada

**DOI:** 10.3201/eid1303.060988

**Published:** 2007-03

**Authors:** Alexander Rodriguez-Palacios, Henry R. Staempfli, Todd Duffield, J. Scott Weese

**Affiliations:** * University of Guelph, Guelph, Ontario, Canada

**Keywords:** Clostridium difficile, bovine, zoonosis, ribotyping, humans, meat, beef, toxinotype III 014/077/027/NAP1, dispatch

## Abstract

*Clostridium difficile* was isolated from 12 (20%) of 60 retail ground meat samples purchased over a 10-month period in 2005 in Canada. Eleven isolates were toxigenic, and 8 (67%) were classified as toxinotype III. The human health implications of this finding are unclear, but with the virulence of toxinotype III strains further studies are required.

*Clostridium difficile* is an important spore-forming human pathogen associated with serious enteric diseases worldwide (*1*–*3*). Recently, the epidemiology of *C*. *difficile*–associated diarrhea (CDAD), appears to have changed; increased illness and relapse rates have been reported (*1*,*3*). Much of this change has been attributed to the emergence of 1 toxigenic strain, classified according to PCR as ribotype 027/toxinotype III and pulsed-field gel electrophoresis (PFGE) as NAP1 (*2*).

Toxigenic strains of *C*. *difficile* typically produce 2 major toxins, A and B, although a small percentage produce only toxin B (*3*). Certain strains may also produce a binary toxin (known as CDT), whose clinical relevance is under investigation. PCR ribotype 027 strains produce all 3 toxins and have a mutated toxin regulatory gene, *tcdC*, which is thought to be associated with increased toxin production in vitro (*2*).

*C*. *difficile* is also associated with enteric diseases in animals, including horses, dogs, and pigs (*4*,*5*). Recent reports indicating that human and animal isolates are often indistinguishable (*4*,*6*) and that PCR ribotype 027 has been isolated from a dog (*7*) have created concerns regarding potential public health implications. *C*. *difficile*, including PCR ribotype 027 (*4*), has also been isolated from dairy calves, beef calves, veal calves, and adult cattle in Ontario (A. Rodriguez-Palacios et al., unpub. data).

The presence of *C*. *difficile* spores in bovine feces indicates the potential for contamination of retail meat products. Although contamination does not necessarily mean foodborne transmission, the possibility of *C*. *difficile* being a foodborne pathogen should be investigated. We therefore evaluated the prevalence of *C*. *difficile* contamination of retail ground meat samples and characterized the isolates.

## The Study

A convenience sampling scheme was used whereby meat samples (beef, n = 53 and veal, n = 7) were purchased from 5 grocery retailers in Ontario (4 stores, 57 samples) and Quebec (1 store, 3 samples), Canada. The number of meat packages purchased per month was 12, 2, 4, 4, 2, 2, 1, 11, 21, and 1, from January to October 2005, respectively.

*C*. *difficile* were isolated by using *C*. *difficile* culture agar supplemented with *C. difficile* moxalactam norfloxacin (CDMN) and 5% horse blood (CM0601, SR0173E, and SR0048C, Oxoid, Basingstoke, United Kingdom) (*8*). *C*. *difficile* broth was prepared by mixing the ingredients of CM0601, except for the agar, with 0.1% sodium taurocholate (Sigma-Aldrich, Inc., St. Louis, MO, USA). Briefly, 4–5 g of each sample was added to 20 mL of prereduced CDMN broth and incubated anaerobically at 37°C for 10–15 days. Alcohol shock for spore selection was performed by mixing 2 mL homogenized culture broth and 96% ethanol (1:1 [v/v]) for 50 min. After centrifugation (3,800 × *g* for 10 min), the sediment was streaked onto *C*. *difficile* agar. Up to 2 suspected colonies (swarming, rough, nonhemolytic) were subcultured from each plate. *C*. *difficile* was presumptively identified on the basis of Gram stain and detection of L-proline aminopeptidase activity (Pro Disc, Remel, Lenexa, KS, USA) and confirmed by identification of the triose phosphate isomerase gene (*9*).

PCR ribotyping and gene identification for toxins A (*tcdA*) and B (*tcdB*), the binding component of CDT (*cdtB*), and the *tcdC* gene were performed as previously described (*4*,*10*). Toxinotyping of selected isolates was also performed (*11*). Antimicrobial drug susceptibility to metronidazole, clindamycin, levofloxacin, and vancomycin was determined for all isolates by using the E-test method (AB Biodisk, Solna, Sweden) on Mueller-Hinton agar (*12*).

*C*. *difficile* was isolated from 12 (20%) of 60 meat samples; 11 (20.8%) of 53 ground beef samples, and 1 (14.3%) of 7 ground veal samples (Table 1). Duplicate analysis was performed on 4 samples, and isolation of *C*. *difficile* was repeatable.

**Table 1:** Description of 12 *Clostridium difficile* strains isolated from retail ground meat samples in Ontario (n = 11) and Quebec  (n = 1), Canada, 2005

PCR ribotype*	PCR toxin genes†	Meat code	Ground beef product	Month sampled	Date sample processed	Store/brand code‡
077	A^+^B^+^, *cdtB^–^,* classic *tcdC,* toxinotype 0	M01	Regular	Jan	Jun 20	A/1
		M02	Regular	Jan	Jun 20	A/1
M26	A^–^B^–^, *cdtB^–^, tcdC^–^*, nontoxinotypeable	M26	Extra lean	May	Aug 10	A/1
M31	A^+^B^+^, *cdtB*^+^, type B/C *tcdC,* toxinotype III	M31	Regular patties	Aug	Sep 1	B/4
		M38	Lean	Aug	Sep 1	B/4
		M41	Medium	Sep	Sep 6	C/7
		M43	Veal	Sep	Sep 6	B/6
		M44	Lean	Aug	Sep 6	B/4
		M47	Lean patties	Sep	Sep 6	B/4
		M51	Lean patties	Sep	Sep 6	B/1
		M52	Lean patties	Sep	Sep 6	B/4
014	A^+^B^+^, *cdtB*^–^*,* classic *tcdC,* toxinotype 0	M54§	Regular	Sep	Sep 20	D/2

*077 and 014; nomenclature of Anaerobe Reference Laboratory, University of Wales, Cardiff, United Kingdom; M26 and M31 temporary nomenclature based on the PCR typing method of Bidet et al. (*10*). Eleven (91.6%) of 12 isolates were toxigenic. †A, toxic gene *tcdA*; B, *tcdB*; *cdtB*, CDTb gene for the binding segment of the binary toxin; – and + indicate absence or presence of the gene. Classic *tcdC,* gene with no deletions; (≈345 bp); type B/C *tcdC*, gene with an ≈18-bp deletion (*4*). ‡Type of processed/packaged product; code 4, ground beef hamburgers sold as a special offer; other codes represent typical commercial ground beef packages. §Commercial package from Quebec.

PCR ribotyping showed distinct patterns (Table 1, Figure). The most common ribotype, which accounted for 8 (67%) of 12 isolates, was different from any ribotype previously identified in our laboratory among ≈1,500 human and animal isolates. This ribotype, designated M31, had genes for toxins A, B and CDT; an 18-bp deletion in the *tcdC* gene and was toxinotype III. These are all molecular characteristics of PCR ribotype 027; however, ribotype pattern M31 was different from ribotype pattern 027 (Figure). PFGE with *Sma*I indicated that although this strain was distinguishable from prototypic strains NAP1, it had ≈80% similarity and was classified as NAP1 (B. Limbago, pers. comm.).

**Figure:**
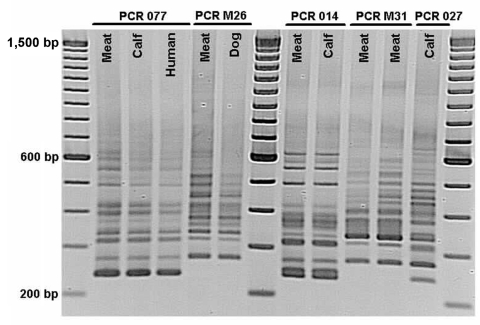
Comparison of PCR ribotypes of *Clostridium difficile* isolates from meat and of human, bovine, and canine origin in Ontario, Canada, 2005, by using the method of Bidet et al (*10*). PCR 077, 014, and 027 represent international ribotype nomenclature recently reported for calves (*4*). PCR M26 and M31 are temporary ribotype designations. Note that PCR M31 and 027, both NAP1/toxinotype III ribotypes, are different.

Two of the remaining 3 ribotypes had classic *tcdC* PCR fragments and did not have the *cdtB* gene. One group (n = 2), classified as PCR ribotype 077/toxinotype 0, had been isolated from calves, dogs, and humans (*4*). Another isolate from Quebec, classified as PCR ribotype 014/toxinotype 0, had also been isolated from calves and humans (*3**,**4*). The fourth isolate, nontoxigenic ribotype M26, had been isolated from dogs (*6*) but could not be toxinotyped because there was no detectable pathogenicity locus (M. Rupnik, pers. comm.). Overall, 3 (25%) of 12 meat *C*. *difficile* isolates were indistinguishable from Ontario human isolates.

All meat isolates were susceptible to metronidazole and vancomycin and resistant to levofloxacin and clindamycin (Table 2). These results are in agreement with previous findings for bovine-derived strains (*4*)

**Table 2:** Drug resistance characteristics of 12 meat-derived *Clostridium difficile* isolates determined by E-test on Muller-Hinton agar after 48 h of incubation*

Antimicrobial drug	MIC_50_, μg/mL	MIC_90_, μg/mL	Range, μg/mL	% Resistant isolates
Vancomycin	0.5	0.75	0.5 to 1.0	0
Metronidazole	0.38	0.5	0.19 to 1.0	0
Levofloxacin	32	32	4 to >32	100
Clindamycin	16.0	24.0	8 to >256	100

*Breakpoints used were vancomycin susceptible, <4.0 μg/mL; vancomycin resistant >32.0 μg/mL; metronidazole susceptible, <8.0 μg/mL; metronidazole resistant, >32.0 μg/mL; clindamycin susceptible, <2.0 μg/mL; clindamycin resistant >8.0 μg/mL; levofloxacin susceptible, <2.0 μg/mL; levofloxacin resistant, >8.0 μg/mL.

## Conclusions

This is the first study to identify *C*. *difficile* spores in retail ground meat intended for human consumption. Previously, a study investigating the role of psychrotrophic clostridia on “blown pack” spoilage of commercial packages of chilled vacuum-packed meats and dog rolls reported 2 incidental isolates of *C*. *difficile* (*13*). More recently, a *C*. *difficile* isolate was identified in a commercial turkey-based raw diet intended for dogs (*14*).

The proportion of meat samples contaminated with *C*. *difficile* in our study (20%, 12/60) seems higher than those in the aforementioned reports. Possible reasons include the use of a more selective culture protocol in this study (*8*) and a potential temporary cluster of isolates with PCR ribotype M31 (Table 1). Those meat samples may have originated from the same larger contaminated batch or were subsequently contaminated at the store level during repackaging of retail products. PCR ribotype M31 was not identified in other samples or stores, which may suggest contamination at the retail level. Because PCR ribotype M31/toxinotype III had not been isolated in our laboratory, contamination during processing is unlikely.

The identification of PCR ribotypes 077 and 014, which are recognized human pathogens (*3*,*15*), is of concern, although the actual risk for disease is unclear. Of additional concern is isolation of toxinotype III strains that have many similarities with PCR ribotype 027, an important cause of CDAD in humans (*2*). This similarity was highlighted by classification of this strain by PFGE as NAP1.

The presence of meat-derived PCR ribotypes indistinguishable from human, bovine, and canine ribotypes further supports the potential risk for cross-transmission among species and suggests that ingestion of viable spores might occur. Although proper cooking of meat is emphasized for reducing the risk for foodborne disease, the fact that *C*. *difficile* is a spore former complicates this issue because spores can survive in ground beef at recommended cooking temperatures (71°C), even when that temperature is maintained for 120 min (A. Rodriguez-Palacios et al., unpub. data).

The clinical and epidemiologic relevance of these microbiologic findings remains unknown. The isolation of *C*. *difficile* from meat samples does not necessarily mean that CDAD is a foodborne disease. Additional studies are required to determine the prevalence of contamination and its clinical relevance.
